# Andrew Victor Schally: Pioneering Neuroendocrinologist and Architect of Luteinizing Hormone-Releasing Hormone Analogs

**DOI:** 10.7759/cureus.69137

**Published:** 2024-09-10

**Authors:** Miloš Grujić, Marija Živković Radojević, Katarina Janković, Neda Milosavljević

**Affiliations:** 1 Clinical Oncology, University of Kragujevac, Faculty of Medical Sciences, Kragujevac, SRB

**Keywords:** andrew schally, biography, historical vignette, luteinizing hormone-releasing hormone, prostate cancer

## Abstract

Andrew Victor Schally is a pioneering figure in endocrinology and neuroendocrinology, whose work has fundamentally transformed the understanding and treatment of hormone-related disorders and cancer. His research, particularly in the isolation, characterization, and clinical application of hypothalamic hormones, has been instrumental in advancing medical science. Schally’s early life, marked by the adversities of World War II, shaped his resilience and determination, driving him to pursue a career in medical research. His groundbreaking discovery of luteinizing hormone-releasing hormone (LHRH) and its analogs revolutionized the treatment of hormone-dependent cancers, especially advanced prostate cancer, by providing an effective alternative to surgical castration. Beyond LHRH, Schally’s contributions to the development of somatostatin analogs have also had a significant impact on the management of acromegaly and neuroendocrine tumors.

This article reviews Schally’s life and work, emphasizing his contributions to endocrinology, particularly in the context of LHRH and its clinical applications. The review outlines his early life and education, his pioneering research on hypothalamic hormones, and the development of LHRH analogs that have become a cornerstone in the treatment of prostate cancer. Schally's ability to translate basic scientific discoveries into practical therapeutic strategies has earned him numerous accolades, including the Nobel Prize in Physiology or Medicine in 1977. His legacy continues to inspire and guide research in endocrinology and oncology, underscoring the lasting impact of his scientific achievements.

## Introduction and background

**Figure 1:**
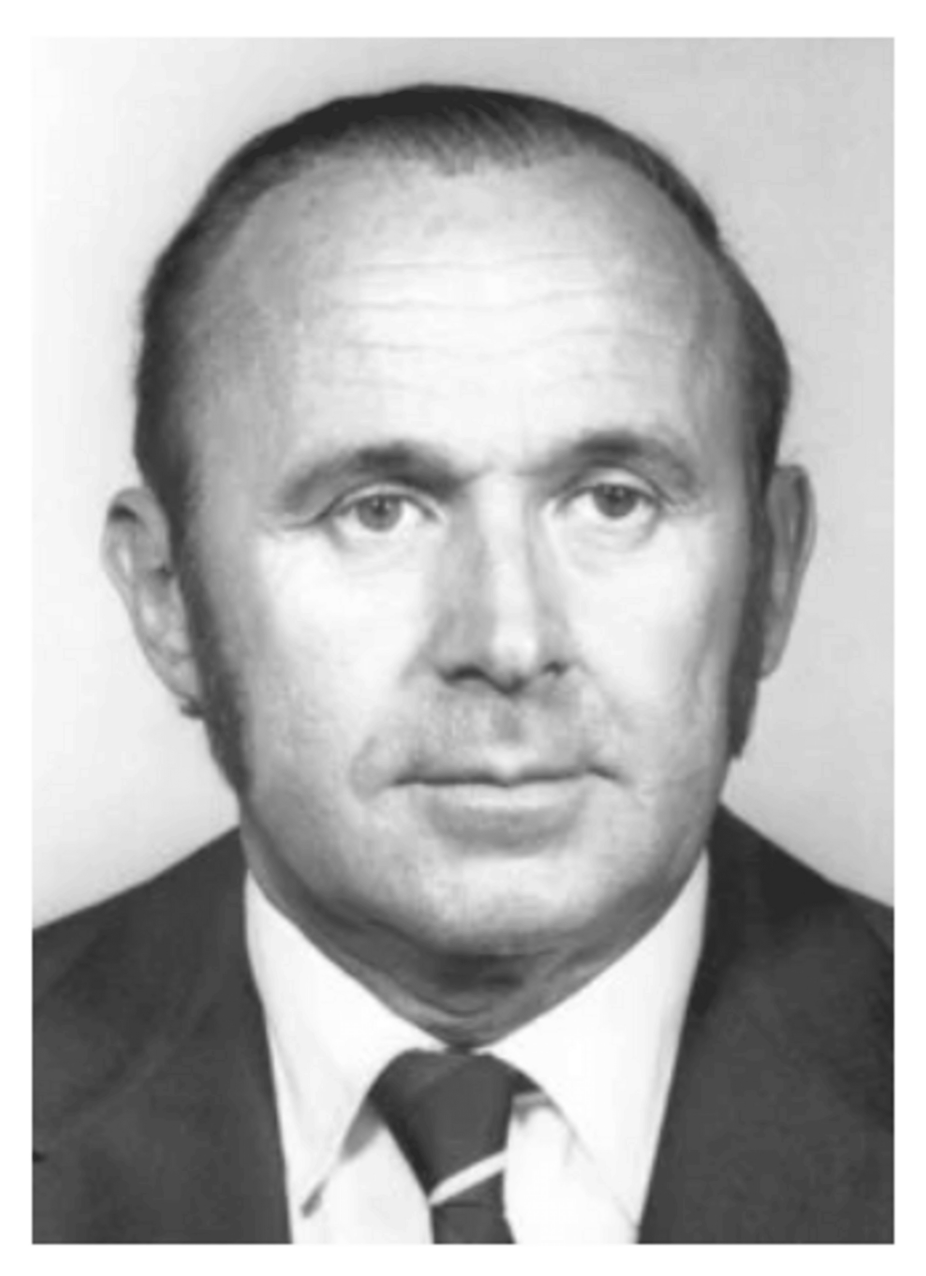
Andrew Victor Schally Image Source: [1]

Andrew Victor Schally (Figure 1) is one of the most influential figures in the fields of endocrinology and neuroendocrinology. His groundbreaking work in the isolation, characterization, and clinical application of hypothalamic hormones has had a profound impact on medical science, particularly in the treatment of endocrine disorders and cancer. Schally’s contributions have revolutionized our understanding of the hypothalamic-pituitary axis and laid the foundation for therapies that have become standard in the management of various conditions, including advanced prostate cancer.

Schally’s early life was marked by adversity. During World War II, he and his family navigated the tumultuous landscape of Eastern Europe, spending time in Romania, Italy, and France. These experiences shaped his resilience and determination, qualities that would later drive his scientific endeavors. After the war, Schally moved to the United Kingdom, where he completed his education and began his journey in medical research [1].

Schally’s scientific career began at the National Institute of Medical Research (NIMR) in London, where he was exposed to the stimulating environment of pioneering scientists, many of whom would go on to receive Nobel Prizes. This formative period ignited his passion for endocrinology, leading him to pursue further studies in Canada at McGill University, where he became deeply involved in the study of the hypothalamus and its role in regulating pituitary function [1,2].

In the 1960s, Schally’s work gained momentum when he moved to the United States and established a laboratory at the Veterans Administration Hospital in New Orleans [1]. It was here that he, along with his collaborators, made significant strides in the isolation of hypothalamic hormones such as thyrotropin-releasing hormone (TRH), luteinizing hormone-releasing hormone (LHRH), and somatostatin [3,4]. His discovery of LHRH and its analogs opened new avenues for the treatment of hormone-dependent cancers, particularly prostate cancer, where LHRH agonists became a cornerstone of therapy [5].

Schally’s innovative research and dedication to translating basic science into clinical applications earned him the Nobel Prize in Physiology or Medicine in 1977, shared with Roger Guillemin and Rosalyn Yalow [6]. His work not only advanced the understanding of neuroendocrinology but also led to the development of new therapeutic strategies that continue to benefit patients worldwide.

This review article will delve into the life and work of Schally, focusing on his contributions to the field of endocrinology, particularly in the development of LHRH analogs and their application in prostate cancer treatment. By exploring his scientific achievements and their impact on modern medicine, we aim to provide a comprehensive understanding of Schally’s enduring legacy.

## Review

Early life and education

Schally was born in Wilno, Poland, in 1926 (now Vilnius, Lithuania). During World War II, he and his family navigated the tumultuous landscape of Eastern Europe, spending time in Romania, Italy, and France, an experience that profoundly influenced his outlook on life and his determination to succeed. In 1945, after the war, Schally moved to the United Kingdom, precisely Scotland, where he completed his high school education and later pursued studies in chemistry in London [1]. There, his interest in science, particularly chemistry, flourished, setting the stage for his future contributions to endocrinology. From a young age, Schally was exposed to a variety of languages, a skill that he honed throughout his life. His linguistic abilities spanned Latin, Romanian, Italian, French, Yiddish, and German. Later, he also became proficient in Spanish and Portuguese. This multilingualism proved invaluable during the 1960s and 1970s, particularly as many clinical trials of LHRH were conducted in Mexico and other Latin American countries. His language skills greatly facilitated his ability to collaborate internationally and deliver lectures across different countries. Despite the hardships of his early life, Schally's experiences and education laid the groundwork for his extraordinary contributions to science [2].

Early research and hypothalamic hormones

At 23, Schally began working at the NIMR in London, where he met and learned from several notable researchers, such as Dr. R. R. Porter, Dr. A. J. P. Martin, and Dr. W. Cornforth, who later received Nobel Prizes [7]. In addition, he had as teachers Sir Charles Harington, who discovered thyroxine, and Dr. R. Pitt-Rivers, who did the same with triiodothyronine [8]. Under the mentorship of renowned scientists, he honed his research skills and developed a keen interest in the hormonal mechanisms that regulate bodily functions. His time at NIMR was crucial in shaping his systematic approach to research and instilling in him a lifelong commitment to the rigorous pursuit of scientific knowledge. This period marked the beginning of his focus on endocrinology, particularly the study of hormones produced by the hypothalamus [1].

In 1952, Schally moved to Montreal, where he joined McGill University and began his pioneering work in endocrinology. It was here that he embarked on his studies of the hypothalamus, the brain region responsible for regulating the endocrine system [2]. At the age of 29, Dr. M. Saffran and his colleagues used in vitro systems in 1955 to detect corticotropin-releasing factor in hypothalamic and neurohypophysial tissues [9]. This groundbreaking work provided the first experimental evidence supporting Dr. G. W. Harris's hypothesis that hypothalamic hormones are key regulators of pituitary function [2]. This period was marked by intensive research, which established Schally as a leading figure in the field and set him on the path to scientific discovery. In 1969, Schally, collaborating with Dr. R. M. G. Nair in New Orleans, successfully determined the accurate amino acid sequence of porcine TRH [3]. This achievement dispelled doubts about hypothalamic research, encouraging them to intensify their focus on LHRH [10].

The discovery of LHRH and its impact

Schally’s work took a significant leap forward in 1957 when he moved to Baylor University, and, in 1962, Schally established a laboratory at the Veterans Administration Hospital in New Orleans, where he led a team focused on the isolation and characterization of hypothalamic hormones [10]. His most significant contribution came in 1971 with the identification of LHRH, a hormone that plays a critical role in the regulation of reproductive functions. He managed to elucidate the full structure of LHRH. After confirming their findings through synthesis, Schally and his team were ready to share their groundbreaking discovery at the Endocrine Society meeting in San Francisco in June 1971. This moment marked a significant milestone in Schally's career, as it resolved a long-standing challenge that had occupied the efforts of many researchers [11].

Clinical applications and the development of LHRH analogs

During the 1970s, Schally developed LHRH analogs, also known as gonadotropin-releasing hormone, which showed promise in treating hormone-dependent conditions [12]. In 1981, he was the first to demonstrate that these analogs could inhibit the growth of prostate cancer in rats, a breakthrough that paved the way for clinical applications [13]. Building on this discovery, Schally, in collaboration with Dr. George Tolis, initiated the first clinical trial of LHRH agonists in 1982, targeting patients with advanced prostate cancer. This pivotal trial confirmed the effectiveness of LHRH agonists in providing palliative care for androgen-dependent prostate cancer [1]. His work demonstrated that these analogs could effectively inhibit the growth of androgen-dependent prostate tumors, leading to their widespread use in clinical practice. Schally also played a key role in the development of sustained delivery systems for these analogs, which are now the preferred method of treatment for advanced prostate carcinoma [12]. Treatment with LHRH agonists provided a non-surgical option that was as effective as orchiectomy but avoided the need for castration, making it a more favorable choice for many patients. Surgical castration (bilateral orchiectomy) is associated with psychological impact, and the perpetuity of this procedure is undesirable for most men [14]. The success of LHRH analogs in oncology paved the way for further research into their potential applications in other areas of medicine.

Contributions to endocrinology and oncology

Beyond LHRH, Schally’s research extended to other areas of endocrinology and oncology. His work on somatostatin analogs has been instrumental in the treatment of acromegaly and neuroendocrine tumors [14]. Schally’s ability to translate basic research into clinical applications has had a lasting impact on the field of medicine, and his contributions continue to influence the development of new therapeutic approaches.

Honors and legacy

**Figure 2:**
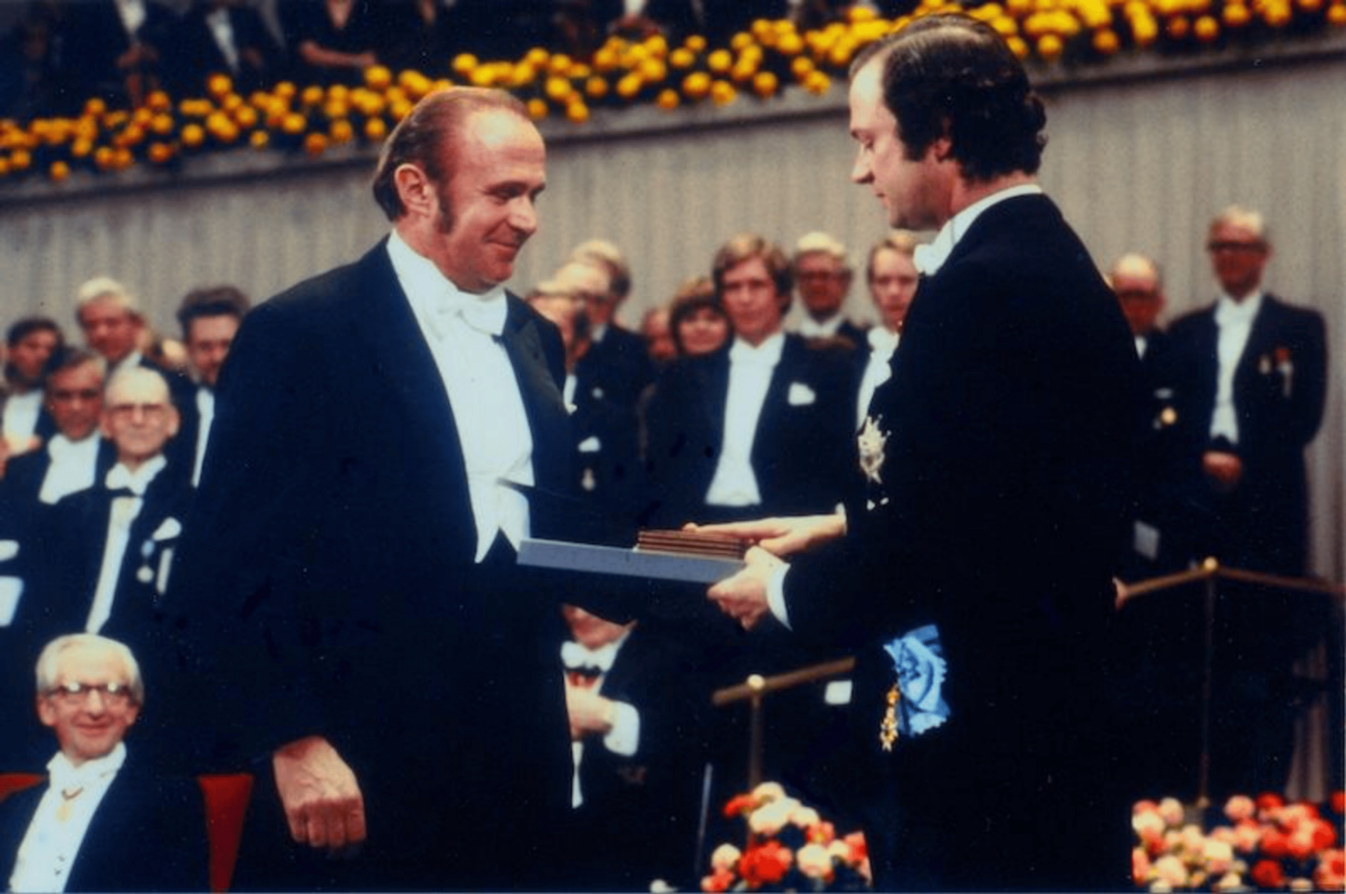
Archive photo of Andrew Victor Schally receiving his Nobel Prize in Physiology or Medicine in 1977 Image Source: [15]

Schally's contributions to science have been recognized with numerous awards, including the Nobel Prize in Physiology or Medicine in 1977 (Figure 2) [1]. He shared this honor with fellow scientists Roger Guillemin and Rosalyn Yalow [6]. His work has had a profound impact on the treatment of endocrine disorders and cancer, and his legacy continues to inspire researchers in the field. Schally's commitment to scientific discovery and his ability to overcome significant challenges throughout his career serve as a testament to his enduring influence in the world of medical research.

## Conclusions

Schally's work on LHRH and its analogs has had a transformative effect on the treatment of prostate cancer and has opened new avenues in the field of neuroendocrinology. His life and career exemplify the power of perseverance, innovation, and a deep commitment to improving human health. Schally’s legacy in medicine will continue to guide future generations of researchers and clinicians in their quest to understand and treat complex diseases.
